# Low Temperature Treatment Affects Concentration and Distribution of Chrysanthemum Stunt Viroid in *Argyranthemum*

**DOI:** 10.3389/fmicb.2016.00224

**Published:** 2016-03-04

**Authors:** Zhibo Zhang, YeonKyeong Lee, Astrid Sivertsen, Gry Skjeseth, Sissel Haugslien, Jihong Liu Clarke, Qiao-Chun Wang, Dag-Ragnar Blystad

**Affiliations:** ^1^The Plant Health and Biotechnology Division, Norwegian Institute of Bioeconomy ResearchÅs, Norway; ^2^State Key Laboratory of Crop Stress Biology for Arid Areas, Key Laboratory of Genetic Improvement of Horticultural Crops of Northwest China, Department of Plant Sciences, College of Horticulture, Northwest A&F UniversityYangling, China; ^3^Department of Plant Sciences, Norwegian University of Life ScienceÅs, Norway

**Keywords:** CSVd, *in situ* hybridization, shoot apical meristem, viroid localization, meristem culture

## Abstract

Chrysanthemum stunt viroid (CSVd) can infect *Argyranthemum* and cause serious economic loss. Low temperature treatment combined with meristem culture has been applied to eradicate viroids from their hosts, but without success in eliminating CSVd from diseased *Argyranthemum*. The objectives of this work were to investigate (1) the effect of low temperature treatment combined with meristem culture on elimination of CSVd, (2) the effect of low temperature treatment on CSVd distribution pattern in shoot apical meristem (SAM), and (3) CSVd distribution in flowers and stems of two infected *Argyranthemum* cultivars. After treatment with low temperature combined with meristem tip culture, two CSVd-free plants were found in ‘Border Dark Red’, but none in ‘Yellow Empire’. With the help of *in situ* hybridization, we found that CSVd distribution patterns in the SAM showed no changes in diseased ‘Yellow Empire’ following 5°C treatment, compared with non-treated plants. However, the CSVd-free area in SAM was enlarged in diseased ‘Border Dark Red’ following prolonged 5°C treatment. Localization of CSVd in the flowers and stems of infected ‘Border Dark Red’ and ‘Yellow Empire’ indicated that seeds could not transmit CSVd in these two cultivars, and CSVd existed in phloem. Results obtained in the study contributed to better understanding of the distribution of CSVd in systemically infected plants and the combination of low temperature treatment and meristem tip culture for production of viroid-free plants.

## Introduction

Viroids are small, single-stranded and infectious RNAs forming a circular structure, and are 246-399 nucleotides in length. Chrysanthemum stunt viroid (CSVd), a member of the family Pospiviroidae (Bouwen and van Zaayen, 2003), can attack several ornamental crops, including *Chrysanthemum* (Bouwen and van Zaayen, 2003), *Argyranthemum* (Zhang et al., 2015), *Dahlia* (Nakashima et al., 2007), and *Petunia* (Verhoeven et al., 1998). Infection by CSVd can result in unmarketable plants and low yield of flowers (Marais et al., 2011; Jeon et al., 2012; Matsushita, 2013; Savitri et al., 2013; Zhang et al., 2015), and possibly to considerable losses. CSVd is also listed as one of the quarantine pathogens in the European Union’s Plant Health Directive (2000/29/EC) (Cho et al., 2013).

Viroids are efficiently transmitted by vegetative propagation of host plants, thus resulting in viroid transmission from generation to generation. Use of viroid-free materials is essential for a sustainable production of vegetatively propagated plants (Zhang et al., 2015). To date, several methods have been developed for production of viroid-free plants, including low temperature therapy (El-Dougdoug et al., 2010; Savitri et al., 2013), thermotherapy (Jeon et al., 2012), leaf primordium (LP)-free shoot apical meristem (SAM) culture (Hosokawa et al., 2004a,b), chemotherapy (Savitri et al., 2013), and cryotherapy (Jeon et al., 2015). Among these, low temperature therapy is most frequently applied.

In low temperature therapy, infected stock materials are treated with low temperatures, for example, 3–8°C, for a certain period. Subsequently, meristems of about 0.2–0.5 mm in size are excised from the low temperature treated materials and cultured for plant regeneration. Frequency of viroid eradication varied among plant species and viroid types (Paduch-Cichal and Kryczyński, 1987; El-Dougdoug et al., 2010). However, the treatment of *Argyranthemum frutescens* plants at 4°C for 6 months, followed by meristem culture and repetition of the combined treatment did not lead to viroid-free plants (Bouwen and van Zaayen, 2003). Reduction of the concentration of CSVd in infected-chrysanthemum grown at low temperature was observed; however, when the plants were transferred to a 30°C glasshouse, the CSVd concentration increased to that of non-treated, infected plants (Chung et al., 2006). Thus, low temperature treatment may decrease viroid concentration and result in larger viroid-free areas in SAM. Viroid localization in low temperature treated shoot tips would provide experimental evidence about effects of low temperature therapy on enlarging viroid-free areas in SAM.

The study of viroid distribution in its host is essential for understanding trafficking and transmission of a viroid. Localization of potato spindle tuber viroid (PSTVd) has been reported in flowers and stems in tomato and *Nicotiana benthamiana* (Zhu et al., 2001; Matsushita et al., 2011). Previously, we found that the ability of CSVd to invade SAMs varied among *Argyranthemum* cultivars (Zhang et al., 2015). However, no systematic localization for CSVd has been done until now.

The objectives of the present study were, therefore, to understand (1) the effect of combined low-temperature treatment and meristem culture on elimination of CSVd from *Argyranthemum*, (2) the effect of low-temperature treatment on CSVd distribution in *Argyranthemum* shoot tips, and (3) the CSVd distribution in flowers and stems of infected *Argyranthemum*.

## Materials and Methods

### Plant Materials

*In vitro* grown ‘Border Dark Red’ and ‘Yellow Empire’ *Argyranthemum* cultivars infected with CSVd were used for a combined low-temperature therapy and meristem culture experiment. Plant materials were cultured on a basic medium (BM) at 23°C under an 18-h light photoperiod with a light intensity of 50 μm m^-2^ s^-1^ provided by cool-white fluorescent tubes (Philips TL-D Super 80, 58W/840). BM was composed of MS (Murashige and Skoog, 1962) supplemented with 0.1 mg/L 1-naphthaleneacetic acid (NAA), 1.0 mg/L benzylaminopurine (BAP) and 0.3 mg/L gibberellic acid 3 (GA_3_), 3% sucrose and 0.6% agar. The pH of the medium was adjusted to 5.8 prior to autoclaving at 121°C for 20 min. Subculture was done every 4 weeks. These *in vitro* stock cultures were used in the following experiments.

*In vivo* grown ‘Border Dark Red’ and ‘Yellow Empire’ infected with CSVd and healthy ‘Border Dark Red’ were used for localization of CSVd in flowers and stems. All stock plants were maintained in net-screened greenhouse conditions, separately.

#### Methods

##### Combined Low-Temperature Therapy and Meristem Culture

Four-week-old *in vitro* grown and CSVd infected ‘Border Dark Red’ and ‘Yellow Empire’ plants were transferred to 5°C under 18-h light photoperiod with a light intensity of 50 μm m^-2^ s^-1^ provided by cool-white fluorescent tubes (Philips TL-D Super 80, 58W/840). After 1, 2, 3, 6 and 12 months, separately, meristems (0.2 mm with 1–2 LP) were taken from low-temperature cultured stocks, and then grown on BM with transfer to new medium every 4 weeks. Shoots (>0.5 cm in length) were developed in about 8 weeks of culture. About 6 months later, plantlets regenerated from meristems were tested for CSVd infection status.

In this experiment, 10 samples were used in each treatment and replicated three times. Regeneration rate was calculated as the percentage of regenerated plants (>0.5 cm in length) after 8 weeks post-culture in the total number of treated plants. CSVd elimination rate was calculated as the percentage of CSVd-free plants in the total number of regenerates tested for CSVd after at least 6 months post-culture. All the CSVd testing was conducted with nucleic acid hybridization assay first and negative samples were confirmed by reverse transcription polymerase chain reaction (RT-PCR).

##### Molecular Diagnostic Techniques

###### CSVd nucleic acid hybridization assay

Chrysanthemum stunt viroid nucleic acid hybridization assay kit (Agdia, USA) was used to screen CSVd in the regenerates following the manufacturer’s instructions. Test samples (0.1 g) were taken from 6-month-old regenerates after meristem culture, and the remaining tissue culture was sub-cultured on new BM. Then the testing tissues were grounded with 150 μl of Ames buffer, centrifuged briefly and 75 μl of supernatant were transferred into a new tube. The supernatant was incubated at 37°C for 15 min, and an equal volume of research grade chloroform was added in a fume hood, followed by shaking, vortex, or inverting. The tubes were briefly centrifuged to separate the contents into aqueous (top) and chloroform (bottom) layers. Finally, 3 μl of the aqueous layers were loaded onto the supplied membrane and the membrane was air-dried before closing the jacket and replacing the membrane in its protective folder. The membrane was sent to Agdia, Inc., USA, for nucleic acid hybridization assay (Podleckis et al., 1993).

###### Detection of CSVd by RT-PCR

Total RNA was isolated from 100 mg of leaf tissue using the Plant RNA Mini Kit (Omega Bio-Tek, USA) following the manufacturer’s instructions. RT-PCR was performed according to Zhang et al. (2014) with CSVd specific primers (forward primer 5′–3′ CGGGACTTACTGTGGTTCC and reverse primer 5′–3′ GGAAGGGTGAAAACCCTGTT).

##### *In Situ* Hybridization of CSVd

Chrysanthemum stunt viroid strand-specific digoxigenin (DIG)-11-UTP labeled probes were synthesized from a recombinant plasmid (PCSVd2) containing a 188 nucleotides fragment, amplified with the forward primer (5′–3′ CGGGACTTACTGTGGTTCC) and the reverse primer (5′–3′ GGAAGGGTGAAAACCCTGTT), of the CSVd genome by *in vitro* transcription (Zhang et al., 2015). *In situ* hybridization was performed according to Zhang et al. (2015). Briefly, ten of each sample, including low-temperature treated shoot tips, flowers and stems of both ‘Border Dark Red’ and ‘Yellow Empire’, were fixed, dehydrated, and paraffin embedded. Ten micrometer thick sections were treated. The hybridization was carried out overnight at 70°C. Then the sections were blocked with blocking solution (Roche, Germany) for 1 h, followed by alkaline phosphatase-conjugated anti-DIG antibody (1:2000 dilution in blocking solution) for 2 h and three times Tris-buffered saline washes. Color reaction was performed using the substrate solution (nitro-blue tetrazolium chloride/5-bromo-4-chloro-3-indolyphosphate *p*-toluidine salt, NBT/BCIP; Promega, USA) in the dark. Results were examined with a light microscope (Leica, Germany).

## Results

### Effects of Combined Low-Temperature Treatments and Meristem Culture on Plant Regeneration and CSVd Eradication in ‘Border Dark Red’

After different periods of low temperature treatment, plants were regenerated from ‘Border Dark Red’ and ‘Yellow Empire’ following meristem culture. Regeneration rates of ‘Border Dark Red’ varied from 47 to 100%, and that of ‘Yellow Empire’ varied from 37 to 87% (**Table 1**). In one special case, after 3 months 5°C treatment, the regeneration rate was 83% (25/30) for ‘Border Dark Red’, while only 11 among 25 regenerated plants could be used for CSVd testing; all the remaining 14 regenerated plantlets stopped growing when they were about 1 cm long. All the regenerated plants from the different treatments were screened for CSVd with nucleic acid hybridization assay; We found that two out of 212 regenerated plants were free of CSVd and both of them were from ‘Border Dark Red’ that were low temperature treated at 5°C for 3 months (**Supplementary Figure S1**, **Table 1**). RT-PCR detection of CSVd confirmed the healthy status (**Supplementary Figure S2**). No CSVd-free ‘Yellow Empire’ was achieved in this experiment.

**Table 1:** Effect of combined low temperature treatment and meristem culture for elimination of CSVd from the infected ‘Border Dark Red’ (BDR) and ‘Yellow Empire’ (YE).

Cultivar	Period of 5°C treatment (month)	Regeneration rate	No. of regenerated plantlets tested	CSVd elimination rate	
				Nucleic acid hybridization assay	RT-PCR
BDR	0	70% (21/30)^a^	21	0	–
BDR	1	100% (30/30)	30	0	–
BDR	2	43% (13/30)	13	0	–
BDR	3	83% (25/30)	11	8% (2/11)^b^	8%
BDR	6	63% (19/30)	15	0	–
BDR	12	47% (14/30)	10	0	–
YE	0	73% (22/30)	13	0	–
YE	1	83% (25/30)	24	0	–
YE	2	37% (11/30)	11	0	–
YE	3	50% (15/30)	15	0	–
YE	6	77% (23/30)	23	0	–
YE	12	87% (26/30)	26	0	–

*^a^Number of the regenerated plants/number of the plants used for meristem culture. ^b^Number of the CSVd-free plants/number of the plants for testing.*

### CSVd-Free Area in the Shoot Tips of ‘Border Dark Red’ was Enlarged After Low Temperature Treatment

**FIGURE 1:**
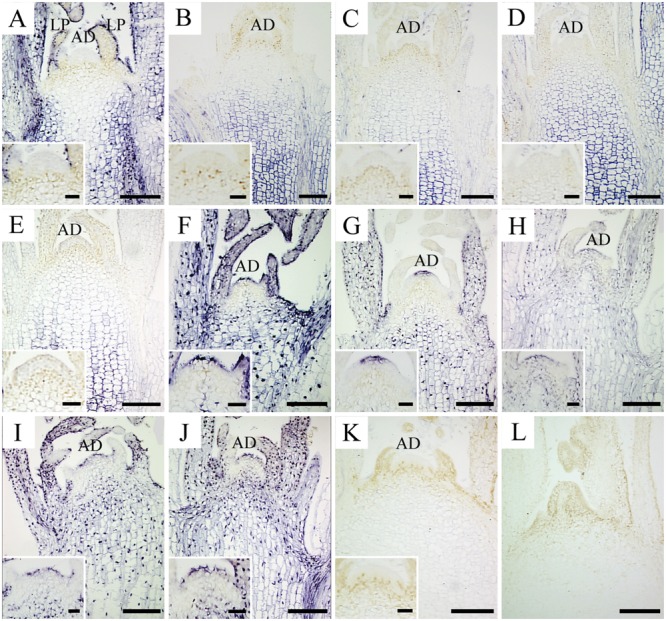
***In situ* localization of CSVd in infected *in vitro* culture *of Argyranthemum* ‘Border Dark Red’ and *A.* ‘Yellow Empire’ after different periods of 5°C treatment. (A)** Shoot apical meristem (SAM) of ‘Border Dark Red’ at room temperature. **(B–E)** SAM of ‘Border Dark Red’ after 1, 2, 3, and 6 months 5°C treatment, respectively. **(F)** SAM of ‘Yellow Empire’ at room temperature. **(G–J)** SAM of ‘Yellow Empire’ after 1, 2, 3, and 6 months 5°C treatment, respectively. **(K)** SAM of healthy ‘Border Dark Red’. **(L)** Negative control with no probe. Inserts indicate higher magnification of AD of **(A–K)**. Scale bars: **(A–L)** 200 μm, inserts 50 μm.

*In situ* hybridization with strand-specific DIG-labeled CSVd antisense-probes resulted in formation of purple-blue color reactions (viroid) in the CSVd-infected tissues (**Figures 1A–J**), while no such color reactions were seen in the healthy samples (**Figure 1K**) or in the tissue that had no probe applied (**Figure 1L**), indicating efficient detection of the viroid. In the diseased ‘Border Dark Red’, CSVd was easily detected in the lower parts of apical dome (AD), in the third LP (observed in the longitudinal section) and in elder tissues of SAM at room temperature; no viroid was detected in the upper most part of AD (**Figure 1A**). The viroid-free area of AD in ‘Border Dark Red’ was approximately 0.2 mm in size. After 1, 2, 3, and 6 months low temperature incubation, CSVd was detected in the elder tissues of SAM, but not in the first four LPs that could be observed in longitudinal sections (**Figures 1B–E**). CSVd was hardly detected in phloem (**Figures 1B–E**). The viroid-free area of AD in ‘Border Dark Red’ was approximately doubled in size after low-temperature treatment. In the healthy ‘Border Dark Red’, no CSVd signals were detected (**Figure 1K**). In CSVd-infected ‘Yellow Empire’, intense viroid signals were revealed throughout SAM, including phloem, elder LPs, even the first two cell layers in AD and the youngest LPs at room temperature (**Figure 1F**). After low temperature treatment for 1, 2, 3, and 6 months, CSVd was still detected in the whole SAM (**Figures 1G–J**), and the distribution pattern was similar to tissues kept at room temperature (**Figure 1F**).

### CSVd was Detected in Flowers and Stem Tissues

**FIGURE 2:**
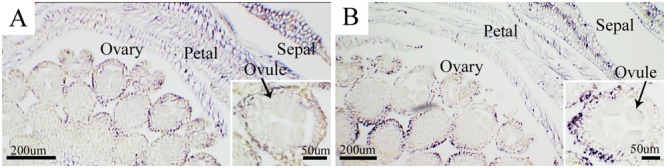
***In situ* hybridization of CSVd in flowers of *Argyranthemum* cultivars. (A)** Cross-section of flower of CSVd-infected ‘Border Dark Red’. Insert indicates higher magnification of ovary. **(B)** Cross-section of flower of CSVd-infected ‘Yellow Empire’. Insert indicates higher magnification of ovary. Scale bars are 200 μm. Inserts: 50 μm.

*In situ* hybridization was carried out to identify CSVd in flower organs of the diseased ‘Border Dark Red’ and ‘Yellow Empire’. CSVd hybridization signals were detected in sepals, petals, and ovaries, but not in ovules (**Figures 2A,B**).

**FIGURE 3:**
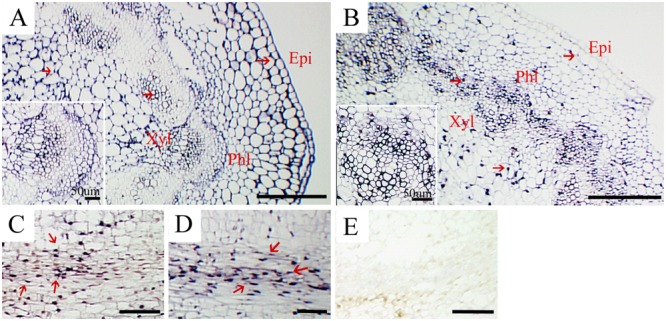
***In situ* hybridization of CSVd in stems of *Argyranthemum* cultivars. (A,B)** Cross sections of stems of CSVd-infected ‘Border Dark Red’ and ‘Yellow Empire’. Inserts indicate higher magnification. **(C)** Longitudinal sections of ‘Border Dark Red’ stems. **(D)** Longitudinal sections of ‘Yellow Empire’ stems. **(E)** Longitudinal sections of healthy ‘Border Dark Red’ stems. All are applied with anti-sense probe; Red arrows indicate CSVd localization. epi, epidermal cells; phl, phloem; xyl, xylem. Scale bars: **(A,B)** 310 μm, **(C–E)** 100 μm.

*In situ* hybridization to locate CSVd in stems of the diseased ‘Border Dark Red’ and ‘Yellow Empire’ was performed. CSVd was readily detected across all tissues in stems, including epidermal cells, pith, xylem, and phloem (**Figures 3A,B**). Detection of CSVd in phloem of ‘Border Dark Red’ (**Figure 3C**) and ‘Yellow Empire’ (**Figure 3D**) indicated that long-distance movement of CSVd occurred in phloem. No CSVd was detected in phloem of healthy ‘Border Dark Red’ (**Figure 3E**).

## Discussion

*In situ* localization of CSVd in stems of ‘Border Dark Red’ and ‘Yellow Empire’ in this study demonstrated that CSVd RNAs are present in epidermal cells, piths, xylem, and phloem. CSVd travels long distance through the phloem of the infected host plants. We also found CSVd could invade SAM of *in vitro* grown ‘Border Dark Red’, including elder tissues and LPs, but not AD, and CSVd can invade throughout the whole SAM of ‘Yellow Empire’, including the AD, which is the same as *in vivo* grown plants (Zhang et al., 2015).

Our study of CSVd invasion of flower organs of ‘Border Dark Red’ and ‘Yellow Empire’ showed that CSVd is present in sepals, petals, and ovaries, but not in ovules. Zhu et al. (2001) found that PSTVd could invade sepals, ovaries, and stamens, but not petals or ovules. Both CSVd and PSTVd are members of the family Pospiviroidae, while invasion abilities can vary with the host range and viroid species. Thus, study of each single viroid species is of importance in order to fully understand the viroid characteristics. It is not clear whether seed can transmit CSVd, Monsion et al. (1973) and Kryczynski et al. (1988) presented contrary reports to Hollings and Stone (1970). Seed transmission requires that the virus or viroid infects the ovules and/or pollen (Wang and Maule, 1994; Zhu et al., 2001; Liu et al., 2014). The current study found CSVd invading the ovaries, but not ovules, which indicates that CSVd is unlikely to be transmitted through seeds in these two *Argyranthemum* cultivars.

Low temperature treatment combined with meristem culture has been successfully applied to eradicate viroids from the host plants: PSTVd from potato (Lizárraga et al., 1980; Paduch-Cichal and Kryczyński, 1987), CSVd (Paduch-Cichal and Kryczyński, 1987; Savitri et al., 2013), and chrysanthemum chlorotic mottle viroid from chrysanthemum (Paduch-Cichal and Kryczyński, 1987). However, there have been no reports on successful elimination of CSVd from *Argyranthemum* until now. In our study: two CSVd-free ‘Border Dark Red’ were obtained after 3 months of low temperature treatment at 5°C and meristem culture, while no CSVd-free ‘Yellow Empire’ plant was acquired in the same experiment. These results indicated that low temperature treatment has some effect on eradicating the viroid from host plants, while the elimination rates varied among viroid species, host species, and even different cultivars of the same host. Our studies of localization of CSVd in *Argyranthemum* shoot tips after low temperature treatment at 5°C shed light on how CSVd distribution is influenced by low-temperature treatment, and explained why CSVd elimination efficiency was quite low in ‘Yellow Empire’.

During low temperature treatment up to 6 months, the CSVd-free area in SAM had become enlarged in ‘Border Dark Red’. Three possible explanations could account for the CSVd distribution changes in ‘Border Dark Red’. (i) The existence of a surveillance system, most likely temperature-dependent (Qu et al., 2005), regulates the selective entry of RNAs into the SAM, which has been documented (Foster et al., 2002; Qi and Ding, 2003; Rodio et al., 2007). (ii) Studies of Pospiviroidae members, such as PSTVd (Owens and Diener, 1982; Branch et al., 1988), cucumber pale fruit viroid (Mühlbach and Sanger, 1979) and citrus exocortis viroid (Semancik and Harper, 1984; Flores, 1989; Rivera-Bustamante and Semancik, 1989), suggested that the replication systems of these viroids involved RNA polymerase for transcription and elongation, RNase for cleavage, and RNA ligase for circularization (Flores et al., 2015). RNA incubation temperature plays a role in RNA synthesis (Diamond and Kirkegaard, 1994; Parkin et al., 1996; Ackermann and Padmanabhan, 2001), and low temperature (like 5°C) could inhibit enzyme activity at variable levels, thus viroid accumulation could be influenced resulting in a larger viroid-free area in the SAM. (iii) Under low temperature stress conditions, oxidative stress can be induced resulting from the production and accumulation of reactive oxygen species (ROS; Suzuki and Mittler, 2006; Luo et al., 2011; Baek and Skinner, 2012). ROS are able to damage cellular components including nucleic acid (Blokhina et al., 2003; Luo et al., 2011). It is possible that the gathered ROS can influence CSVd accumulation. The reason that low temperature has no effect on the CSVd distribution pattern in SAM of ‘Yellow Empire’ is still unclear. After meristem culture of low-temperature treated ‘Border Dark Red’, only two CSVd-free plants were obtained after 3 months 5°C treatment, while no viroid-free plant was obtained after six and even 12 months of treatment at 5°C, which is unexpected based on *in situ* hybridization of CSVd. It is possible that current *in situ* hybridization protocols are not sensitive enough to detect very low level of CSVd in the low temperature-treated tissues. Zhu et al. (2001) have also mentioned detection limitation of *in situ* hybridization; however, until now *in situ* hybridization is the most sensitive method to localize viroids inside plant tissues.

## Conclusion

Chrysanthemum stunt viroid is a viroid that can infect *Argyranthemum* and cause serious economic loss. Low temperature can enlarge the CSVd-free area in the shoot tips of ‘Border Dark Red’ and increase viroid-elimination efficiency after meristem culture. Additionally, the effect of low temperature on CSVd distribution in *Argyranthemum* shoot tips is dependent on the cultivars of the host. Localization of CSVd in flowers and stems of systematically infected *Argyranthemum* is essential for better understanding of distribution and transmission of CSVd and other viroids.

## Author Contributions

ZZ, Q-CW, and D-RB designed the study. ZZ performed ISH, RT-PCR experiments and wrote the manuscript. AS, GS, and SH performed meristem culture, collected data, and CSVd testing. YL, Q-CW, JLC and D-RB revised and finalized the manuscript. All authors read and approved the final manuscript.

## Conflict of Interest Statement

The authors declare that the research was conducted in the absence of any commercial or financial relationships that could be construed as a potential conflict of interest.

## Supplementary Material

FIGURE S1 **Nucleic acid hybridization assay of CSVd in regenerated plants after combined low temperature treatment and meristem culture.** Controls A–H, dilution series of CSVd-positive control in 1:2 ratio; Neg, a known CSVd-free plant as negative control; A1–H9, different regenerated plants.

FIGURE S2 **RT-PCR detection of CSVd in regenerated plants after combined low temperature treatment and meristem culture.** M: 100 DNA ladder; P: CSVd infected ‘Border Dark Red’ (positive control); MQ, Milli-Q water; N: Healthy ‘Border Dark Red’ (negative control); B1 and B2: CSVd free ‘Border Dark Red’ tested with CSVd nucleic acid hybridization assay.

## Abbreviations

AD: apical dome
CSVd: chrysanthemum stunt viroid
DIG: digoxigenin
LP: leaf primordium
PSTVd: potato spindle tuber viroid
RT-PCR: reverse transcription polymerase chain reaction
SAM: shoot apical meristem
