# Supplementary figures and images for: Low Temperature Treatment Affects Concentration and Distribution of Chrysanthemum Stunt Viroid in Argyranthemum
Source: Front Microbiol. 2016 Mar 4;7:224. doi: 10.3389/fmicb.2016.00224 (PMC4777735; doi:10.3389/fmicb.2016.00224)

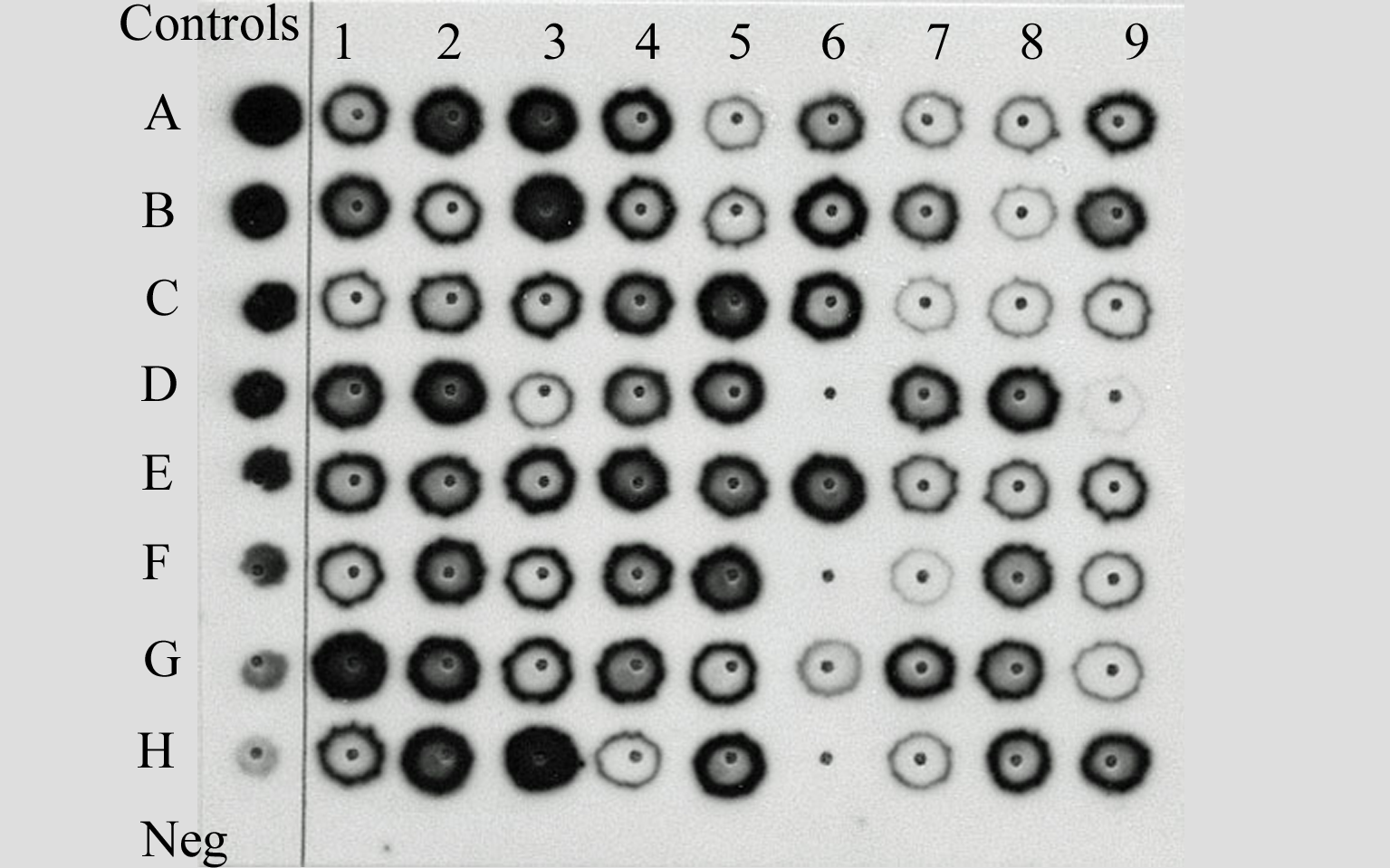

Supplement: FIGURE S1 — Nucleic acid hybridization assay of CSVd in regenerated plants after combined low temperature treatment and meristem culture. Controls A–H, dilution series of CSVd-positive control in 1:2 ratio; Neg, a known CSVd-free plant as negative control; A1–H9, different regenerated plants. [file Image_1.TIF]

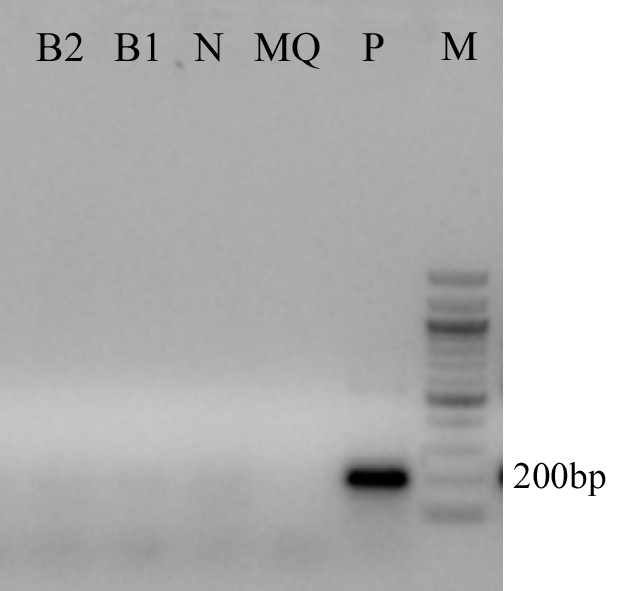

Supplement: FIGURE S2 — RT-PCR detection of CSVd in regenerated plants after combined low temperature treatment and meristem culture. M: 100 DNA ladder; P: CSVd infected ‘Border Dark Red’ (positive control); MQ, Milli-Q water; N: Healthy ‘Border Dark Red’ (negative control); B1 and B2: CSVd free ‘Border Dark Red’ tested with CSVd nucleic acid hybridization assay. [file Image_2.TIF]
